# Contraindications to immunotherapy: a global approach

**DOI:** 10.1186/s13601-019-0285-4

**Published:** 2019-09-11

**Authors:** C. Pitsios, M. Tsoumani, M. B. Bilò, G. J. Sturm, P. Rodríguez del Río, R. Gawlik, F. Ruëff, G. Paraskevopoulos, E. Valovirta, O. Pfaar, M. A. Calderón, P. Demoly

**Affiliations:** 1 Medical School, University of Cyprus, P.O. Box 20537, 1678 Nicosia, Cyprus; 2 Division of Infection, Immunity and Respiratory Medicine, School of Biological Sciences, University of Manchester and NIHR Manchester Biomedical Research Centre, Manchester University Hospitals NHS Foundation Trust, Manchester Academic Health Science Centre, Manchester, UK; 3 Dept. of Internal Medicine, Allergy Unit, University Hospital, Ancona, Italy; 4 Dept. of Dermatology and Venereology, Medical University of Graz, Graz, Austria; 5 Allergy Outpatient Clinic Reumannplatz, Vienna, Austria; 6 Allergy Section, Children’s University Hospital “Niño Jesús”, Madrid, Spain; 7 Dept. of Internal Medicine, Allergy and Clin. Immunology, Silesian University of Medicine, Katowice, Poland; 8 Dermatology and Allergology Clinic and Policlinic, Ludwig-Maximilians University, Munich, Germany; 9 Allergy Outpatient Clinic, 401 General Military Hospital of Athens, Athens, Greece; 10 Terveystalo Turku, Allergy Clinic, University of Turku, Turku, Finland; 11 Department of Otorhinolaryngology, Head and Neck Surgery, Universitätsmedizin Manneim, Medical Faculty Mannheim, Heidelberg University, Mannheim, Germany; 12 Center for Rhinology and Allergology, Wiesbaden, Germany; 13 Section of Allergy and Clinical Immunology, Imperial College London, National Heart and Lung Institute and Royal Brompton Hospital NSH, London, UK; 14 Département de Pneumologie et Addictologie, Hôpital Arnaud de Villeneuve, University Hospital of Montpellier, Montpellier, France; 15 UMR-S 1136, IPLESP, Equipe EPAR, Sorbonne Université, 75013 Paris, France

**Keywords:** Allergen immunotherapy, Venom hypersensitivity, Contraindications, Beta-blocker, Asthma, Autoimmunity, Malignancy, ACE-inhibitor, Pregnancy

## Abstract

**Background:**

Recommendations on contraindications to allergen immunotherapy (AIT) have been independently developed by National and International Societies/Academies. AIT contraindications are mainly based on case reports, case-series, or experts’ opinion, while evidence-based information is limited. The aim of the present review was to describe existing guidelines on contraindications to AIT and to highlight differences between them.

**Main body:**

An extended review of the literature regarding contraindications to AIT for respiratory allergy and venom hypersensitivity was performed. Furthermore, Societies and Academies registered in the World Allergy Organization and EAACI databases, were asked for additional information. Only AIT guidelines published under official auspicies were included. A large heterogeneity among the various recommendations on contraindications was registered. Common contraindications to most of the guidelines were: lack of adherence, pregnancy before the start of AIT, the use of beta-blockers, certain age groups, uncontrolled asthma, autoimmune diseases and malignancies.

**Conclusion:**

As new data arise, revisions might soon be needed allowing AIT in the cases of patients treated with ACE inhibitors and beta-blockers, in elderly patients and in patients with concomitant autoimmune diseases and neoplasias in remission. The decision to prescribe AIT is always tailor-made, balancing risk vs benefit. Creating globally accepted guidelines would help Allergologists in their decision making.

## Background

Allergen immunotherapy (AIT) is an evidence-based efficacious treatment option for respiratory and venom allergy, however, there are some concomitant diseases and underlying conditions that emerge as safety limitations and lead to contraindications to AIT [1, 2]. Several controversies exist on these contraindications, like whether they are justified or not and their distinction in ‘absolute’ or ‘relative’. Furthermore contraindications are different for subcutaneous airborne AIT (SCIT), for sublingual AIT (SLIT) and for subcutaneous venom immunotherapy (VIT). As a result of these controversies, there are clinical, legal and ethical issues that often arise [1].

Due to ethical and practical reasons, it is not always possible to perform clinical trials on AIT’s contraindications. Most of the existing studies regarding this topic are observational case-series or case-reports and only few evidence-based information regarding contraindications to AIT exist [1]. The decision to use AIT in patients with a contraindicated condition or concomitant disease is often based on risk–benefit balance; AIT may be justified in individual cases that are expected to be benefited more than posed to potential risk.

Guidelines on AIT, describing contraindications, have been developed and published by international academies and national societies of allergology and clinical immunology and are mainly based on experts’ opinion [1–17]. In the frame of an EAACI Task Force, a position paper on contraindications has been published and the recent EAACI’s Guidelines on Immunotherapy are in accordance with it [18, 19].

The present review has been produced by the EAACI Task Force on “Contraindications to Immunotherapy” and aims to offer a global overview on the topic revealing the differences of the different guidelines and existing contradictions.

## Method

### Search strategy

Guidelines, consensuses and position papers pertaining to AIT (for airborne allergens and Hymenoptera venoms) were retrieved from electronic bibliographic databases (Pubmed, Cochrane library, Google scholar). *Specific immunotherapy*; *allergen immunotherapy*; *venom immunotherapy*; *sublingual*; *subcutaneous*; *guidelines and contraindications* have been the individual search words for this research. The composite search terms were (allergen OR venom) AND immunotherapy AND (guidelines OR contraindication).

In cases that the search resulted in multiple guidelines from a single Society/Academy, the most recent one was preferred. The web sites of national academies and/or societies registered in the WAO and EAACI databases have been searched for official guidelines on AIT (and contraindications), in order to confirm that the most recent ones have been retrieved, or to obtain unpublished ones [20].

Furthermore, officially appointed contact-persons/webmasters were approached by email, when the access to the official websites was allowed to members only. Not all webmasters replied and consequently an effort to contact directly the National Committees was made. Articles in various languages have been retrieved and translated into English, with the help of the respective national societies.

### Inclusion/exclusion criteria

Guidelines that have been prepared and published under official auspicies of Societies and/or Academies that are members of WAO and/or registered in the WAO and EAACI databases, were included. Contraindications to AIT were searched for, including SLIT, SCIT and VIT.

Contraindications reported in guidelines of other non-allergy Societies/Colleges were excluded. Same for guidelines of Regional societies, when National guidelines where published. Reviews reported as “experts’ opinion”, that were not specified as official guidelines of a Society/Academy of allergology/immunology, were also excluded.

### Categorization of contraindications

In order to provide a uniform list of the suggested contraindications, an effort was made to minimize the heterogeneity of the various terms regarding the same disease/condition, merging them to more generic ones. However, when particular mentions on well-specified diseases or conditions had been made, these were listed separately from the generic terms. The use of the terms ‘absolute’ and ‘relative’ was not always clarified and in some guidelines other descriptive words had been used to replace them. Two reviewers (MT, CP) made the distinction of these terms in each guideline. Any discrepancies were resolved through discussion and, if necessary, a third reviewer was consulted (PD).

### Data synthesis

A table including all diseases/conditions that have been described as contraindications and the relative Societies/Academies, was created by MT, reviewed by CP and PD (Table 1). In the case that specific guidelines on different types of AIT have been retrieved, they are mentioned separately in Table 1.

**Table 1:** Conditions and comorbidies considered as contraindications to AIT, by Allergy Societies/Academies (in alphabetical order) and year of publication

	AAAAI-2011	ASCIA-1997	BSACI (SCIT)-2011	BSACI (VIT)-2011	CMICA-2011	DSA-2005	EAACI (SCIT)-2015	EAACI (SLIT)-2015	EAACI (VIT)-2015	FSACI-2011	German-speaking-2014	IFIACI (SCIT)-2014	IFIACI (VIT)-2014	JSA-2017	NSAI-2011	PTA (SLIT)-2016	SEIAC-2011	SFA-2003	SFFA-2009	WHO-1998
Lack of communication	A			A		A	R	R	R	A	A	A	A		R	A		A	A	R
Start during pregnancy	R^a^	R	R	R	R	R	A	A	A	R	A^a^	A^b^	R	A	R	R	R	R	R	R
Beta-blockers	R	A	A	R	R	A^c^	R	R		A^c^	A	R	R	A	R	A	R	A	R	R
ACE-inhibitors	R			R	R				R		A^d^								R	
Antidepressants				R																
Children < 5-6 yo		R	A		R		R^e^	R^e^	R^e^			A^b^	A		R		R	A	R^b^	R^c^
Elderly > 65 yo					R												R			
Cardiovascular diseases	R		R		A	A	R	R		A^c^				R	R	A			A	R
Uncontrolled asthma	R	R	A	A	A	A	A	A	A	A	A^g^	A	A	A	R	A	R	A	A	R
Co-existing active diseases			A		A	A	R	R	R			R			R	A				
Immunodeficiency			R	R	R	A	R	R	R	A	A	A			R	A	R	A	A	R
Autoimmunity		R	R	R	R	A	R^f^	R^f^	R^f^	R	R	A	R	A	R	A	R	A	A	R
Immunosuppressive agents			R				R	R	R		A	A						A		
Chronic infections						A	R	R	R	A			R			A			A	
Malignancy		R	R	R	R	A	A	A	R	A	R	A	R	A	R	A		A		
Dermatitis		R				R				R					R				R	R
Temporary conditions											A	R				R	R			
Inadequate means/training		A		A																
Anaphylaxis to AIT		A																		

Acronyms of the Societies/Academies are explained in the “Abbreviations” section

A: absolute contraindications or contraindications of major importance

R: relative contraindications or special precautions/considerations

^a^VIT can be initiated during pregnancy in isolated cases of life-threatening insect venom allergy

^b^SCIT is contraindicated while SLIT is not

^c^Not a contraindication for VIT

^d^Contraindicated for VIT

^e^For children < 2 yo it consists an absolute contraindication

^f^For autoimmune disorders in active form it consists an absolute contraindication

^g^Following the German NVL (Nationale VersorgungsLeitlinie) for the definition of partially controlled asthma, it is mentioned that “AIT may be performed in children in case of partially controlled astma—provided they rarely experience asthma symptoms”

## Results

### Guidelines’ retrieval

**Fig. 1:**
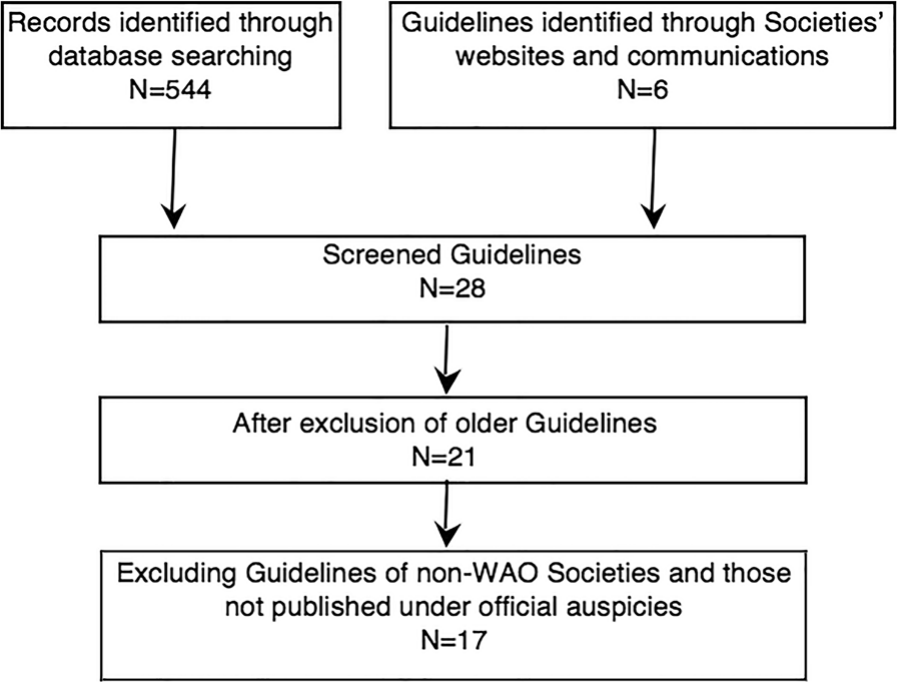
Flow diagram of selection of guidelines on contraindications to AIT

A total of 544 publications was the outcome of the online research. 51 National and 4 International Societies/Academies were contacted. Twenty-eight suitable papers have been retrieved; some of them have been published in official journals of national societies, not included in the main medical search engines. The exclusion of older guidelines of Societies, keeping the renewed ones, reduced the suitable papers to 21. Although published in the Official journal of CSACI by members of its Board and followed by allergists, Canadian guidelines were not included because they do not consist official ones (personal communication) [21]. The inclusion criteria reduced the number of papers on contraindications from 21 to 17 (Fig. 1).

### Contraindication terms’ selection

A variety of contraindications was found, as described in Table 1. An heterogeneous use of terms has been used to describe the grade of contraindication and it was tried to reduce them to “absolute” or “relative”. The terms “special considerations”, “temporary precautions” and “reviewable” have been replaced by “relative”, while the term “of major importance” has been replaced by “absolute” [2, 5].

### Guidelines to contraindications; the big picture

Most of the Academies/Societies do not include official guidelines regarding contraindications to AIT in their websites. Some of them have accepted and reproduce official guidelines of other societies/academies, e.g. SLAAI refers to the AAAAI/ACAAI/JCAAI and CMICA guidelines, while HSACI follows both AAAAI and EAACI ones. In some countries, like Argentina, there are different guidelines; AAAeIC uses WHO guidelines, while SAAeI uses CMICA and WHO guidelines (personal communication). Societies from German-speaking countries (Germany, Austria and Switzerland) have developed common guidelines [14].

Most guidelines refer to the administration of AIT with airborne allergens. In most Societies/Academies there are comments referring to the treatment of venom allergy and some contraindications don’t apply as absolute for VIT. British and Italian Societies have developed special guidelines for Hymenoptera venom allergic patients [4, 22, 23].

Contraindications for SLIT are expressed in the position papers of German-speaking countries and of EAACI, while distinct guidelines have been developed in Poland by PTA [1, 14, 17].

### Concomitant diseases and conditions described as contraindications

**Fig. 2:**
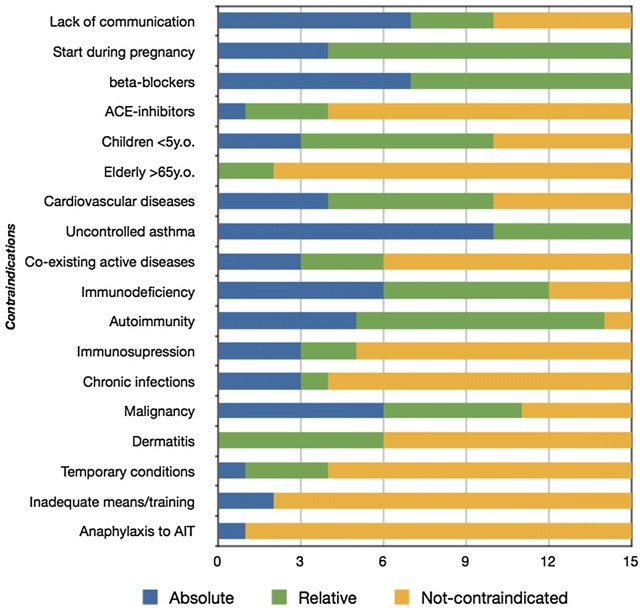
Conflicting guidelines on whether a condition/comorbidity consists absolute, relative or no contraindication to SCIT

A large heterogeneity among guidelines was noticed, however it is commonly accepted that: lack of communication (and/or cooperation), pregnancy before the start of AIT, treatment with beta-blockers, certain age groups, uncontrolled asthma, immune diseases and malignancies are included in most guidelines as (absolute or relative) contraindications to initiate AIT (Fig. 2) [1–17]. Inadequate medical means and AIT performed by clinicians without relative training are reported as absolute contraindications by ASCIA, also mentioning former anaphylaxis to AIT as a contraindication to continue [15]. Transient interfering situations (acute febrile illness, inflammatory and infective diseases, exacerbation of asthma) are mentioned by IFIACI under the term “temporary contraindications” [11]. Gastrointestinal inflammation, dental extraction or oral surgery and infections have been designated as temporal contraindications for SLIT [8, 14, 17].

Initiation of AIT is contraindicated during pregnancy, while AIT can be continued if the woman became pregnant after starting it [1–17]. AAAAI guidelines for pregnancy mention that initiation of VIT might be considered in high-risk conditions, while in the case that a “patient is receiving a dose unlikely to be therapeutic, discontinuation of immunotherapy should be considered” [2]. Danish guidelines suggest to stop AIT when pregnancies’ complications occur, or the patient expresses the slightest hesitation [9].

As far as drugs are concerned, cardiovascular drugs are of main consideration. In most guidelines it is suggested to replace beta-blockers (even when administered topically, e.g. eye drops) and/or ACE-inhibitors, with equally efficacious alternative drugs [1, 2, 4, 10, 12, 14]. EAACI guidelines include beta-blockers as relative contraindication for SCIT and SLIT and ACE-inhibitors as relative ones for VIT [1]. BSACI guidelines mention the use of tricyclic antidepressants as a contraindication, suggesting their replacement by selective serotonin reuptake inhibitors, before commencement of VIT [4]. On the other hand the use of MAO-inhibitors, is currently very limited so the risk of their co-administration with epinephrine has elapsed.

Cardiovascular and lung diseases are comorbidities suggesting special considerations since they might result in impaired tolerance of hypotension, and bronchospasm, responding poorly to resuscitation and emergency treatment if AIT-induced anaphylaxis occurs [2–4, 7–10, 12–14]. Asthma is highlighted in all guidelines and patients with severe asthma, with persistent symptoms remaining uncontrolled despite optimal pharmacological treatment and FEV1 < 70% of the predicted value, are ruled out [1–4, 6–11, 14]. A convergence on severe/uncontrolled/unstable asthma is noticed in the guidelines. German guidelines mention that AIT “may be performed in children in case of partially controlled asthma—provided they rarely experience asthma symptoms” [14].

Age limits were commented in most AIT guidelines recording infancy as a main concern (lower limit: 5 or 6 years of age in different guidelines) [1–3, 5–7, 10, 13]. In CMICA guidelines, SCIT is indicated in children above 2 years old [7]. AIT has also been mentioned as a contraindication in elderly, because of high incidence of comorbid medical conditions and frequent use of the aforementioned cardiovascular drugs [6, 10].Concurrent depletion of the immune system in the cases of primary and secondary immunodeficiency syndromes, use of immunosuppressive agents, autoimmune diseases and chronic infections are mentioned as contraindication in most guidelines, though there is no solid evidence that AIT is actually harmful in these patients [3–7, 9–14]. AIT is also contraindicated for patients with concomitant lymphoid malignancies and malignant tumors in general [3–7, 9–14]. In the article cited in SFA official website, a special reference appears suggesting to consider AIT in patients with healed tumors [5]. Regarding patients with HIV disease, AIT is contraindicated only at the AIDS stages [1, 2, 19].

## Discussion

Contraindications are developed in order to exclude patients with comorbid diseases to which AIT might constitute an aggravating co-factor, or who would not respond adequately to the treatment of anaphylaxis. Under some circumstances, an individual ‘risk–benefit’ analysis should be performed by specialists and AIT might be suggested even in high-risk patients (‘relative contraindications’). As regards to the terms absolute and relative, their interpretation is often difficult, albeit they are useful to highlight that the decision to treat with AIT is not always a black and white issue. Hymenoptera venom allergy is potentially fatal, so VIT is considered a treatment option even when comorbidities exist.

There are data from retrospective (SCIT and VIT) and randomized controlled (SLIT) studies proving the safety of continuing AIT during pregnancy [24]. There are scarce data on the initiation of SCIT and SLIT during pregnancy reporting no maternal or fetal complications [25, 26], while initiation of AIT has also been reported by a limited number of allergists in CONSIT survey, without major problems [27]. Major problems were rarely noticed (1.2%) by allergists who continued VIT in pregnant women [28]. In opinion surveys most responders would continue AIT (all types) during pregnancy [35, 36], would not start SCIT [27, 29] and don’t consider the start of SLIT during pregnancy as a contraindication [27].

Based on old experimental studies (performed in humans and animals) and on surveillance surveys (regarding fatalities), the concomitant use of β-blockers is thought to constitute a risk factor for more severe and treatment-resistant anaphylaxis [30–34]. The use of ACE-inhibitors is also considered a risk-factor for serious hypotension in the case of allergic reactions during AIT, but caution is based on few case-reports [35]. Recently, with mouse model, it has been shown that the combined administration of β-blockers and ACE-inhibitors exacerbates anaphylactic symptoms, synergically increasing FcεRI-mediated mastcell histamine release [36].

Real-life studies have provided data suggesting that β-blockers and ACE-inhibitors don’t appear to increase the incidence of systemic reactions during VIT and SCIT [27, 35, 37–40]. SCIT has been associated with lower incidence of myocardial ischemia and infarction, compared with conventional therapy, so VIT is highly suggested in patients with cardiovascular diseases [41]. The presence of cardiovascular diseases in patients that are under β-blockers and ACE-inhibitors is a confounding factor for side-effects during AIT, so large prospective observational studies on the safety of these medicines, taking under consideration the underlying diseases, are needed [42].

Regarding lower age limits it has been suggested that AIT can be initiated in preschoolers (less than 5 years of age) if indications exist [1, 2, 7]. Performing SCIT and SLIT to toddlers has been proved to be safe and the prompt diagnosis of a systemic reaction in young children is easy to assess [43, 44]. However, SCIT in infancy might be dangerous due to young children’s inefficiency to communicate the symptoms pointing at the onset of anaphylaxis. Physicians have been discouraged from practising it in very young patients, due to limited published evidence supporting its benefit.

Although epidemiological studies support a decline of the prevalence of allergic diseases in elderly, there are immunological data suggesting that type-2 cytokines pattern is not defective in older age [45–47]. Even though debated, late-onset allergy to airborne allergens should be treated with AIT, taking under consideration concomitant diseases that pose contraindications. Benefits of AIT include protection when cardiovascular diseases coexist (as underlined in the case of VIT) [41], but also the reduced risk of side effects to the chronic use of corticosteroids (diabetes, osteoporosis, hypertension etc.) and anti-histamines (sedation and anti-cholinergic effect) [45].

Safety and effectiveness of AIT require adherence to the treatment and an adequate patient-physician collaboration. Psychiatric disorders are an heterogeneous group of mental health conditions. In the case that patients’ ability to report symptoms suggestive of anaphylaxis has been impaired or a psychiatric condition that affects adherence to the treatment exists, AIT is contraindicated [48, 49]. Collaboration with the caregiver and the psychiatrist, can increase adherence [50]. The heterogeneous symptomatology of psychiatric disorders should be taken into consideration and tailor-made decisions should be offered to patients. On the other hand non-adherence due to repeated forgetfulness or negligence (even in the absence of the aforementioned disorders) is a common problem in medicine, affecting safety and efficacy of AIT [51]. Continuing AIT is contraindicated to nonadherent patients, although no specific definitions in AIT guidelines have described which these limits are [1, 2, 14].

There is a gap of evidence connecting AIT to an effect on autoimmune disorders. A paradox is the apparently beneficial use of honeybee stings for the treatment of rheumatoid arthritis, suggested by Acupuncture medicine [52], while VIT is contraindicated in patients with the same concomitant disease [1, 3–19]. The outcome of a large nationalwide study from Denmark, analyzing data over a decade, was that patients treated with AIT, receiving aluminium-containing allergen preparations, had lower incidence of autoimmunity compared to those on conventional treatment [41]. The evidence that AIT or the contained adjuvants can trigger or deteriorate autoimmune diseases in mainly based on a limited number of anecdotal case reports [53, 54]. It would also be useful if guidelines were differentiating between practicing AIT with concomitant organ specific autoimmune disorders (Hashimoto thyroiditis, rheumatoid arthritis etc.) and systemic autoimmune disorders; German guidelines have done so [14].

The need to reassess neoplastic diseases for high-risk venom allergic patients, is also mentioned by guidelines [1, 11, 14]. Epidemiologic association between allergies and IgE levels (total and specific) with lower risk of developing certain malignancies has been expressed [55]. Although strong proof is missing, interfering with Th2 immunity may effect cancer; low dose (1 and 3 μg/mL) of recombinant Der p 2 can enhance in vitro cell motility and invasiveness of non-small cell lung cancer cells, promoting metastatic ability of carcinoma cells [56]. On the contrary there are some data showing that upregulation of IgG4 antibodies offers protection from malignant melanocytes, so hypothetically their upregulation during AIT might benefit the cure of melanoma [57].

Defining contraindications to AIT is useful for allergists, though some of them require further clarification. As shown by CONSIT and by AAAAI’s surveys, experienced allergists often use AIT beyond contraindications, on an individual basis [27–29]. CONSIT concluded that prescribing SLIT or performing VIT is less avoided when a relative contraindication occurs, and this is due to the high safety profile of SLIT or to the risk–benefit ratio favouring VIT [28]. However, AIT treatment in these cases should only be performed after thoroughly informing and training patients and their concordance is warranted.

There are ethical and legal conflicts on performing evidence-based clinical trials on patients with concomitant diseases. However, performing prospective surveillance studies by registering data of patients that are treated after giving informed consent can provide more concrete data. Such a study is currently contacted on the use of β-blockers and ACE-inhibitors, with preliminary results favouring treatment with AIT (personal communication). In the case of contraindications, the decision to prescribe AIT weighs risk vs benefit; decision is easier to be made in the case of VIT. The field of VIT contraindications can be investigated for most of relative contraindications and results may provide useful data that can apply to SLIT and SCIT. Regarding SLIT, since it is a safer alternative to SCIT, contraindications that apply may soon me minimized, given that long-term registration of case-series can be created.

## Conclusions

A major heterogeneity regarding contraindications resulted from the present systematic review of the current literature in the field. Most guidelines are not evidence-based and reproduce older ones or copy each other. As AIT is evolving, novel forms of AIT are being produced and new data are arising, there is a profound need to update contraindications regularly. Ideally a globally accepted consensus on contraindications to AIT should be published aiming to reach international harmonization in this specific important domain of AIT. We believe that the present work paves the ground for such a future task.

## Data Availability

Not applicable.

## Abbreviations

AAAAI: American Academy of Allergy Asthma and Immunology
AAAeIC: Argentine Association of Allergy & Clinical Immunology
ACAAI: American College of Allergy Asthma and Immunology
ÄDA: Association of German Allergists
ASCIA: Australasian society of Allergy and Clinical Immunology
BSACI: British Society for Allergy & Clinical Immunology
CMICA: Mexican College of Clinical Immunology and Allergy
CSACI: Canadian Society of Allergy & Clinical Immunology
DGAKI: German Society for Allergology and Clinical Immunology
DSA: Danish Society of Allergology
EAACI: European Academy of Allergy & Clinical Immunology
FSACI: Finnish Society of Allergology and Clinical Immunology
GPA: Society of Pediatric Allergology and Environmental Medicine
HSACI: Hellenic Society of Allergology and Clinical Immunology
IFIACI: Federation of Italian Societies of Immunology, Allergology and Clinical Immunology
JCAAI: Joint Council of Allergy Asthma and Immunology
JSA: Japanese Society of Allergology
NSAI: Norwegian Society of Allergology and Immunopathology
ÖGAI: Austrian Society for Allergology and Immunology
PTA: Polish Society of Allergology
SAAeI: Argentine Society of Allergy and Immunopathology
SEAIC: Spanish Society of Allergy and Clinical Immunology
SFA: French Society of Allergology
SFFA: Swedish Association for Allergology
SLAAI: Latin American Society of Allergy, Asthma & Immunology
SSAI/SGAI: Swiss Society of Allergology and Immunology
WAO: World Allergy Organization
